# An Intensive Care Outbreak Caused by *Burkholderia cepacia* from Bacterial Filters

**DOI:** 10.3390/pathogens14030266

**Published:** 2025-03-08

**Authors:** Özlem Aytaç, Elif Seren Tanrıverdi, Ömür Gündağ, Feray Ferda Şenol, Gülden Eser Karlıdağ, Barış Otlu

**Affiliations:** 1 Medical Microbiology, Elazığ Fethi Sekin City Hospital, 23280 Elazığ, Türkiye; 2 Department of Medical Microbiology, Faculty of Medicine, Inonu University, 44000 Malatya, Türkiye; 3 Infectious Diseases and Clinic Microbiology Department, Elazığ Fethi Sekin City Hospital, 23280 Elazığ, Türkiye; 4 Infectious Diseases and Clinical Microbiology Clinic, Elazığ Fethi Sekin City Hospital, Health Sciences University, 23280 Elazığ, Türkiye

**Keywords:** *Burkholderia cepacia*, outbreak, AP-PCR, hospital infection

## Abstract

Background: We report a hospital outbreak caused by *Burkholderia cepacia* that occurred in 16 patients admitted to intensive care units in Elazığ, Türkiye, between 19 March and 23 April 2024. Methods: The outbreak investigation was initiated on 23 March 2024, four days after *B. cepacia* was detected in four different patients. Environmental samples were collected from various parts of the hospital to find the source of the outbreak. Arbitrarily Primed Polymerase Chain Reaction (AP-PCR) was performed to determine the genetic relationship between environmental and patient samples. Results: In total, 16 of 18 *B. cepacia* isolates were obtained from tracheal aspirate culture. A total of 10 of 16 patients developed hospital-acquired pneumonia due to *B. cepacia*. Among the environmental cultures in the intensive care units, only the respirator bacterial filter grew. The isolate obtained here was in the same cluster as the isolate obtained from patient samples, resulting in a dominant clustering rate of 94.4%. Conclusions: Improper and inappropriate use of respirators and equipment can lead to outbreaks. Early detection of the outbreak, identification of the source, and taking appropriate measures quickly to contain the outbreak are key.

## 1. Introduction

*Burkholderia cepacia complex* (BCC) is a group of obligate aerobic, catalase-positive, non-glucose-fermenting, Gram-negative bacilli including at least 22 known closely related species [1]. They are commonly found in natural environments such as water and soil. They can be found as phytopathogens or biocontrol agents in plants. In humans, they generally do not cause disease since the airway is cleared by mucociliary activity in immunocompetent individuals. However, *Burkholderia cepacia complex* has been identified as a human pathogen that can cause many infections leading to significant morbidity and mortality as opportunistic pathogens [2,3,4,5]. It is a very important pathogen especially in terms of affecting immunocompromised patients such as chronic granulomatous lung disease, Cystic Fibrosis, and patients with lung diseases that cause a decrease in lung function [6].

In studies, BCC has been associated with healthcare-associated outbreaks caused by contamination of medical devices, antiseptic solutions, parenteral and nebulized drugs, and other environmental sources [7,8,9,10].

A comprehensive study in 2019 documented 125 *Burkholderia cepacia*-related hospital-acquired infection outbreaks worldwide between 1970 and 2019. A total of 3287 patients were affected in these outbreaks. In 74.4% of the outbreaks (93 outbreaks), sources of contamination were identified as medicine bottles, disinfectants, and antiseptics. In 76% of the outbreaks (95 outbreaks), effective prevention and control measures were implemented. Only 33 reports indicated that a combination of patient, personnel, and environmental measures were used [11].

In a study conducted in Turkey, a *Burkholderia cepacia* outbreak caused by contaminated foam soaps in a hospital intensive care unit was investigated. The outbreak was detected by a significant increase in *B. cepacia* in blood cultures. Measures such as increased hand hygiene, contact isolation, and environmental disinfection were implemented to control the outbreak, and the outbreak lasted approximately 270 days [12].

Nosocomial pneumonia is an infection acquired after a hospital stay of at least 48 h. Lung infections that occur in people connected to mechanical ventilation devices in hospitals are called ventilator-associated pneumonia (VAP). VAP typically affects critically ill patients in the intensive care unit (ICU) who have been on mechanical ventilation for at least 48 h [12,13,14].

Early-onset pneumonias in intensive care patients are those that occur between the 48th and 72nd hours of mechanical ventilation, while late-onset pneumonias are those that occur after the 72nd hour. Early-onset pneumonias are seen to occur due to aspiration of pathogens colonized in the oropharynx during intubation. In late-onset pneumonia, resistant microorganisms (*MRSA*, *Acinetobacter* spp., *Pseudomonas aeruginosa*) are involved, and high mortality is mentioned in infections developing with these agents [15]. There are defined risk factors and comorbidities for these common bacteria such as age, gender, underlying diseases (malignancy, cerebrovascular diseases, cardiovascular, COPD, diabetes, acute and liver disease, and chronic renal failure), hospitalization period, immunosuppressive treatments, invasive procedures, surgery, parenteral blood transfusion, burn or trauma, nutrition, and antibiotic use. Risk factors, such as growth of resistant microorganisms in lower respiratory tract cultures of patients with ICU, hospitalization for ≥28 days, malignancies, living in nursing homes or long-term care facilities, chronic granulomatous diseases, primary immune deficiency states, corticosteroid use, and cystic fibrosis have been defined [16]

Here, we describe a hospital outbreak caused by *Burkholderia cepacia* in a tertiary care center in Elazığ, Türkiye, between 15 March and 23 April 2024. The outbreak occurred in patients hospitalized in intensive care units (ICUs) with and without structural lung disease. The outbreak occurred in patients admitted to intensive care units (ICUs) with and without structural lung disease and was diagnosed as nosocomial pneumonia.

## 2. Materials and Methods

### 2.1. Hospital Settings

This study was conducted at Türkiye, Elazığ, a tertiary hospital with a total of 1038 beds and 187 intensive care beds. The outbreak occurred in various intensive care units. Our intensive care beds have an average occupancy rate of 85–90%. In our hospital’s intensive care unit, patients with respiratory distress and respiratory failure requiring oxygen support, patients with hypotension requiring support, patients in shock, sepsis, patients with multiple trauma requiring close vital monitoring (traffic accidents inside and outside the vehicle, falls, etc.), and patients with drug and chemical toxicity are generally admitted. The Centers for Disease Control and Prevention (CDC) definitions were used for the diagnosis of hospital-acquired infections (HAIs) [17]. The hospital infection control team performs active surveillance in all intensive care units in the hospital. Infection control team nurses and physicians work with the intensive care unit and microbiology team to detect HAIs. Clinical and active surveillance cultures are checked daily, and the infection control team organizes daily rounds in the ICU, conducting outbreak investigation in collaboration with the microbiology department.

The study was approved by the institutional review board and the entire study protocol was in accordance with the Declaration of Helsinki. The preparation of the text followed the guidelines for Outbreak Reports and Response Studies of Nosocomial Infections.

**Figure 1:**
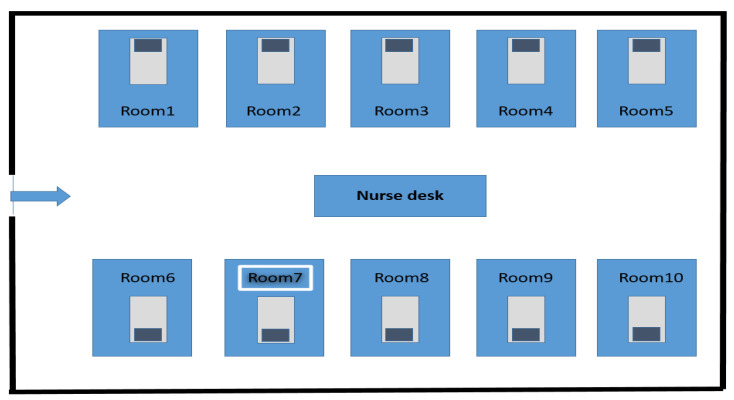
Our BCC positive case and intensive care unit architecture, bed layout.

Our BCC positive case and the intensive care unit architecture and bed arrangement are shown in Figure 1.

### 2.2. Environmental Investigation

On 23 March 2024, four days after the first detection of the *Burkholderia cepacia complex*, investigations of possible sources began. Samples were collected from various departments, including commonly used commercial products in infected patients, distilled water, chlorhexidine and alcohol solutions, povidone iodine, chlorhexidine mouthwash solution, heparin, drinking water, tap water, sinks, drain holes, frequently touched surfaces, respiratory equipment bacterial filters, cleaning rooms, cleaning equipment and carts, medical equipment (thoracic drainage aspirators, electrocardiogram, portable X-ray device, portable cassettes, ECMO water heaters), hemodialysis device, dialysis wastewater, and the hands of staff.

Sterile cotton swabs were used to collect the samples. Sampling was supervised and managed by a trained infection control committee nurse.

### 2.3. Microbiological Analysis and Antibiogram

Samples obtained from patients and the environment were cultured according to traditional culture methods, and culture plates were incubated at 35–37 °C in an environment containing 5–10% CO_2_ for 18–24 h for those with growth and up to 96 h for those without growth, and growth was evaluated at the end of incubation.

Eosin methylene blue (EMB) (RTA, Kocaeli, Türkiye), 5% sheep blood agar (RTA, Kocaeli, Türkiye), and chocolate agar (RTA, Kocaeli, Türkiye) were used as media. Selective agar for BCC was not used. Colony types were identified by VITEK-MS^®^ system (bioMérieux SA, Marcy l’Etoile, France). Antimicrobial susceptibility for BCC was performed using the Kirby Bauer disk diffusion method for trimethoprim-sulfamethoxazole, ceftazidime, meropenem (BIOANALYSE, Ankara, Türkiye) and the gradient diffusion method for levofloxacin in accordance with the Clinical and Laboratory Standards Institute (CLSI) (2023) criteria [18]. Bacterial samples were stored at −80 °C until molecular analysis.

### 2.4. Molecular Analysis

All isolates were reseeded on EMB(RTA,Kocaeli,Türkiye) and 5% sheep blood agar (RTA,Kocaeli,Türkiye) for molecular analysis. Appropriate samples with culture growth were analyzed by arbitrarily primed polymerase chain reaction (AP-PCR) in the Molecular Microbiology Laboratory of the Department of Microbiology, Malatya Turgut Ozal Medical Center.

### 2.5. Arbitrarily Primed Polymerase Chain Reaction (AP-PCR)

The M13 primer (5′-GAG GGT GGC GGT TCT-3′) was used for genotyping the isolates by Arbitrarily Primed Polymerase Chain Reaction (AP-PCR). The AP-PCR method was applied as previously described by Durmaz et al. [19]. The thermal cycling program, shown in Table 1, was used in a thermal cycler to amplify the DNA regions specific to the M13 primer using the prepared PCR mixture.

After amplification, 12 µL of the amplification product was mixed with bromophenol blue at a ratio of 3:4 and subjected to electrophoresis in 1.5% agarose gel at 100 V for 250 min. The band profiles of the strains were photographed using the Gel Logic 2200 imaging system (Kodak Co., Rochester, NY, USA) and compared. Band profile analysis was performed using the GelCompar II software system (version 6.6; Applied Maths, Sint-Martens-Latem, Belgium). Dice correlation coefficients were used for similarity calculations, and the Unweighted Pairwise Grouping Mathematical Averaging (UPGMA) method was used for cluster analysis.

Isolates with similarity coefficients greater than 85% were considered to belong to the same or related clones, while those with similarity below 85% were classified as distinct or unrelated clones.

## 3. Results

A total of 18 BCC isolates were detected between 23 March and 23 April 2024. Of the 18 isolates, 7 were from female patients, 10 were from male patients, and 1 was a bacterial filter belonging to the patient. The median age of the patients was 72 years (ranging from 20 to 92 years). Of the 18 isolates, 16 were isolated from tracheal aspirate culture and one from cerebrospinal fluid (CSF) culture. One isolate was obtained from the bacterial filter of the mechanical ventilation device of a patient hospitalized in anesthesia intensive care unit (ICU). Summarizes the patients’ mean number of days of hospital stay before and after bacterial isolation, infection status, and response to treatment (Table 2).

The first BCC isolate was isolated from the tracheal aspirate sample of a patient diagnosed with multiple myeloma and receiving mechanical ventilation support due to respiratory distress in the internal medicine ICU of our hospital. Including the first case detected, a total of 17 cases were detected from the chest ICU, neurology ICU, and anesthesia ICUs. Sixteen of the cases with BBC growth were patients receiving mechanical ventilation support and growth was detected in tracheal aspirate samples of these patients. BCC was accepted as the cause of pneumonia by the infection control committee in 10 of the 16 patients in whom BCC was detected and who received mechanical ventilation support.

No growth was detected in repeated cultures obtained from possible sources. Only 1 of the 18 isolates we obtained was obtained from the bacterial filter of the mechanical ventilation device, and this isolate was included in the other 17 clonally identical isolates.

**Figure 2:**
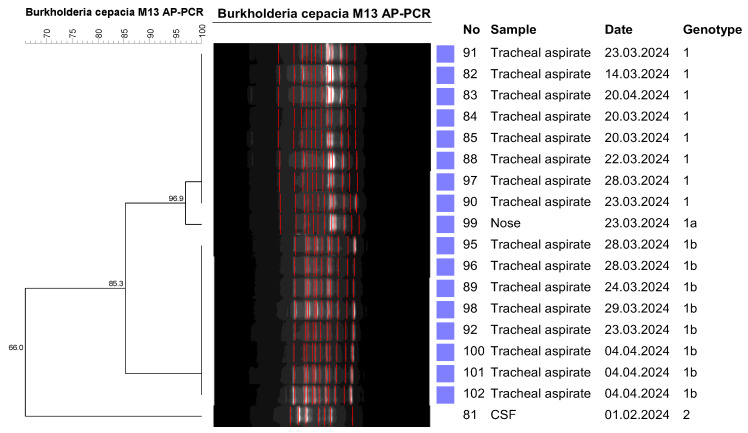
AP-PCR patterns of *Burkholderia cepacia* isolates.

AP-PCR analysis of the 18 *Burkholderia cepacia* isolates included in the study revealed two different genotypes. Of the 18 *Burkholderia cepacia* isolates, 17 were clonally identical and formed a single cluster, resulting in a dominant clustering rate of 94.4%. The clonally distinct isolate was a CSF sample, which was taken while the patient was hospitalized in a different hospital (Figure 2). No BCC isolate was subsequently detected in the hospital to which the patient from whom the CSF sample belonged was referred.

The results of antimicrobial susceptibility tests showed that all *B. cepacia* isolates associated with the outbreak were susceptible to meropenem, levofloxacin, and trimethoprim-sulfamethoxazole and resistant to ceftazidime.

## 4. Discussion

By revealing the clonal relationship between bacteria, epidemic isolates are separated from sporadic or endemic isolates, outbreak-associated strains are identified, and information about the source, reservoir, and scope of the outbreak can be obtained [20]. AP-PCR is a classical variation of PCR and is based on amplifying unknown genomic regions using random primers [21].

Defining healthcare-associated infections is essential for clinical risk management plans as well as for attributing the burden to healthcare facilities in the event of harm [22].

An increase in the number of identified infections above the initial rate is defined as an outbreak. Identifying correlations between sources of contamination and specific pathogens can provide valuable guidance to public health researchers, clinicians, infection preventionists, and hospital epidemiologists in determining the source of an outbreak [23]. It is believed that examining the relationship between strains during surveillance programs after determining that there is an outbreak and identifying the causative agent plays an important role in understanding the dynamics of contamination and evaluating the effectiveness of the preventive strategies (maintenance measures and disinfection treatment) implemented [24].

AP-PFGE revealed the same strain (94.4% similarity) causing the outbreak. Although we could not clearly identify the source, growth was detected in the bacterial filters of the respirator used in the intensive care unit. In addition, due to the lack of BCC isolation as a result of the increased frequency of changing the bacterial filter and breathing circuit, it is thought that the outbreak was a result of the bacterial filter and breathing circuit being contaminated by long-term use without changing. It was thought that the first case in which we detected bacteria was due to external contamination of the breathing circuit used for a long time and the bacterial filter in which we detected growth. It was also observed that the frequency of cleaning of the breathing circuit was low. Although we could not detect growth, it was concluded that there was a contamination caused by the materials and personnel used during the cleaning of this circuit. The infection control committee reviewed the measures for nosocomial infection control and took necessary precautions and corrective action. In this context, studies were carried out by the infection control committee regarding determining the source of the outbreak, isolation of infected and colonized patients, hand hygiene and personnel training, medical device and solution controls, environmental cleaning and decontamination, monitoring of antibiotic and antiseptic resistance, and monitoring and reporting of outbreaks within the hospital.

In addition, the infection control committee carried out the following activities:

- Previously, the respiratory circuits were used without change throughout the patient’s hospitalization, but after the outbreak, the respiratory circuits began to be changed when the patients had excessive secretions;
- The respiratory circuits were wiped three times a day;
- It was determined that portable X-ray cassettes were used directly from patient to patient. X-ray cassettes began to be used by putting on protective bags while passing from patient to patient;
- The consultant physician, physiotherapist, dietician, and other personnel who were providing service by visiting all intensive care units at the same time were trained on the use of appropriate gloves, hand hygiene, and infection control measures. Those of these personnel who were not wearing protective gowns were warned to wear them;
- Some intensive care units that did not use bacteria filters constantly were warned about the correct and appropriate use of the filters and their inspections were carried out;
- Dialysis drain covers were open, closed, and disinfection was carried out;
- A detailed room cleaning was carried out after each patient was discharged;
- The humidification rate of humidifiers in the respiratory circuit was reduced to reduce bacterial colonization.

BCC acts as a potential source of nosocomial infections in the hospital setting. This is because BCC has the potential to multiply and survive in the presence of antibiotic solutions, established invasive medical devices, and disinfectants [25]. The virulence of BCC may differ between BCC members as well as within members. Mutant strains have been shown to survive better and be more virulent in human macrophages. In addition, it is known that these bacteria have highly variable antimicrobial susceptibility and are naturally resistant to more than one antibiotic [26]. In our study, levofloxacin, meropenem, cotrimoxazole, and ceftazidime, which are effective antibiotics against BCC, were tested according to the guidelines of the Clinical and Laboratory Standards Institute [27], and it was observed that all isolates were resistant only to ceftazidime. Treatment with trimethoprim-sulfamethoxazole was initiated in patients who were accepted as the causative agent of infection and the treatment was found to be effective. Our first patient, the index case, may have contracted the infection because of immunosuppressive treatment, low immunity, and a bacterial filter that was used without being changed for a long time. The remaining cases may have been exposed to this organism due to inadequate hand hygiene practices, improper cleaning and disinfection procedures, and the use of contaminated ventilation equipment.

The infection control committee monitored the compliance of intensive care unit staff with hand hygiene and provided feedback to healthcare workers. Infection prevention strategies were reminded to staff to prevent hospital acquired infections. The effectiveness of control measures was evaluated after cases by continuous follow-up of cases both clinically and microbiologically. The last case with *B. cepacia* colonization was detected 38 days after the start of the outbreak, and no further clusters were detected after respirators were maintained and bacterial filters replaced. The control measures were considered effective because there were no new cases of BCC pneumonia. Timely notification to the clinician, implementation of infection control measures such as hand hygiene, proper cleaning, and disinfection of respirators and filters, and the cohort of infected cases reduced this outbreak.

Preventing hospital-acquired infections (HAI) is becoming increasingly important due to antimicrobial resistance and emerging pathogens. It is important to review and strengthen effective infection control protocols, which are the basic issues for the management of epidemics, to carry out studies to optimize the use of antibiotics, to establish an active surveillance system and to ensure early diagnosis of new and resistant microorganisms, and to ensure stronger coordination between infectious disease specialists, microbiologists, public health officials, and healthcare professionals.

## Figures and Tables

**Table 1:** Thermal cycling profile used for AP-PCR.

Step	Temperature	Time	Cycles
Denaturation	94 °C	5 min	2 cycles
Primer annealing	40 °C	5 min	
Primer extension	72 °C	5 min	
Denaturation	94 °C	1 min	40 cycles
Primer annealing	40 °C	1 min	
Primer extension	72 °C	2 min	

**Table 2:** Duration of hospital stay before and after bacterial isolation, those considered to be infectious agents, and response to treatment.

Admission	Patients Diagnosis	BFID	AFID	Isolate Site	İF	TR
1	MM, Pneumonia	15	8	Tracheal aspirate	NO	-
2	Cardiac arrest	150	40	Tracheal aspirate	YES	YES
3	Cardiac arrest	103	123	Tracheal aspirate	YES	YES
4	Ischemic SVH	23	37	Tracheal aspirate	NO	-
5	COPD	49	55	Tracheal aspirate	NO	-
6	Parkinson	5	3	Tracheal aspirate	NO	-
7	COPD	64	5	Tracheal aspirate	YES	YES
8	PE	55	15	Tracheal aspirate	YES	YES
9	Traffic accident	6	4	Tracheal aspirate	YES	YES
10	Traffic accident	118	74	Tracheal aspirate	YES	YES
11	SAH	26	8	Tracheal aspirate	YES	YES
12	Traffic accident	3	7	Tracheal aspirate	YES	YES
13	SVD	3	6	Tracheal aspirate	NO	-
14	MI	4	15	Tracheal aspirate	YES	YES
15	Arrest	159	6	Tracheal aspirate	YES	YES
16	PE	10	60	Tracheal aspirate	NO	-

## Data Availability

The datasets generated and analyzed during the current study are not publicly available due to the requirement to protect patient confidentiality.

## Abbreviations

The following abbreviations are used in this manuscript:

AP-PCR	Arbitrarily Primed Polymerase Chain Reaction
BCC	*Burkholderia cepacia complex*
HAI	Hospital-acquired infections
CDC	The Centers for Disease Control and Prevention
ICUs	Intensive care units
EMB	Eosin methylene blue
CLSI	Clinical and Laboratory Standards Institute
UPGMA	Unweighted Pairwise Grouping Mathematical Averaging
CSF	Cerebrospinal fluid.
SAH	Subarachnoid hemorrhage
SVD	Serebrovasküler disease
MI	Myocardial infarction
BFID	Hospital Stay (ICU) before First Isolation (Days)
AFID	Hospital Stay (ICU) after First Isolation (Days)
IF	Infectious Factor
TR	Treatment Response
PE	Pulmonary embolism
MM	Multiple Myeloma
